# Metabarcoding of Fecal Samples to Determine Herbivore Diets: A Case Study of the Endangered Pacific Pocket Mouse

**DOI:** 10.1371/journal.pone.0165366

**Published:** 2016-11-16

**Authors:** Deborah D. Iwanowicz, Amy G. Vandergast, Robert S. Cornman, Cynthia R. Adams, Joshua R. Kohn, Robert N. Fisher, Cheryl S. Brehme

**Affiliations:** 1 U.S. Geological Survey, Leetown Science Center, Kearneysville, West Virginia, United States of America; 2 U.S. Geological Survey, Western Ecological Research Center, San Diego, California, United States of America; 3 U.S. Geological Survey, Fort Collins Science Center, Fort Collins, Colorado, United States of America; 4 University of California San Diego, Division of Biological Sciences, La Jolla, California, United States of America; Oklahoma State University, UNITED STATES

## Abstract

Understanding the diet of an endangered species illuminates the animal’s ecology, habitat requirements, and conservation needs. However, direct observation of diet can be difficult, particularly for small, nocturnal animals such as the Pacific pocket mouse (Heteromyidae: *Perognathus longimembris pacificus*). Very little is known of the dietary habits of this federally endangered rodent, hindering management and restoration efforts. We used a metabarcoding approach to identify source plants in fecal samples (N = 52) from the three remaining populations known. The internal transcribed spacers (ITS) of the nuclear ribosomal loci were sequenced following the Illumina MiSeq amplicon strategy and processed reads were mapped to reference databases. We evaluated a range of threshold mapping criteria and found the best-performing setting generally recovered two distinct mock communities in proportions similar to expectation. We tested our method on captive animals fed a known diet and recovered almost all plant sources, but found substantial heterogeneity among fecal pellets collected from the same individual at the same time. Observed richness did not increase with pooling of pellets from the same individual. In field-collected samples, we identified 4–14 plant genera in individual samples and 74 genera overall, but over 50 percent of reads mapped to just six species in five genera. We simulated the effects of sequencing error, variable read length, and chimera formation to infer taxon-specific rates of misassignment for the local flora, which were generally low with some exceptions. Richness at the species and genus levels did not reach a clear asymptote, suggesting that diet breadth remained underestimated in the current pool of samples. Large numbers of scat samples are therefore needed to make inferences about diet and resource selection in future studies of the Pacific pocket mouse. We conclude that our minimally invasive method is promising for determining herbivore diets given a library of sequences from local plants.

## Introduction

Knowledge of the diet of rare herbivores is essential to providing sound management and habitat restoration recommendations [1–6], as it underlies individual growth rates, health and survivorship, and population stability [7, 8]. Yet this understanding is often difficult and costly to acquire from direct observation, such that researchers often resort to other lines of evidence. Methods that supplement or replace direct observation include microscopic examination of feces, cheek contents, or alimentary canal, plant alkane fingerprints, protein electrophoresis of gut contents, reflectance spectroscopy, and stable isotope analysis [9–12]. While these methods continue to offer important insights, limiting factors can include labor, expert knowledge, instrument throughput, and cost. Methods also vary in resolution and ascertainment bias. For example, besides requiring a highly trained practitioner, microscopy can underestimate the importance of plants with higher digestibility or faster decomposition [13]. Similarly, stable isotope analysis can provide a long-term, integrated view of diet and dietary shifts of a population over time, but rarely allows multiple species to be conclusively identified in the diet and their relative importance determined. More recently, DNA sequencing strategies have become popular, allowing high taxonomic resolution from minimal starting material [11, 14, 15]. However, it is expensive and time-consuming to clone and sequence many independent DNA fragments using traditional means. The development of next-generation sequencing promises to increase the speed and accuracy of genetic dietary analysis, and may be particularly useful for relatively complex mixtures such as feces.

**Fig 1:**
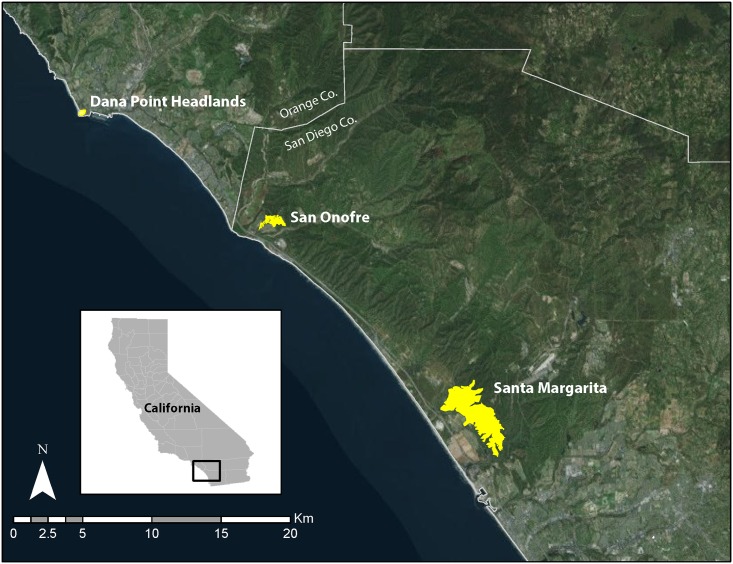
Locations of the three extant Pacific pocket mouse (*Perognathus longimembris pacificus*) populations in southern California. Dana Point Headlands (Dana Point, CA) is the only site located in Orange County, California. Both Santa Margarita and San Onofre (both within the bounds of Marine Corps Base Camp Pendleton [MCBCP]) are found in San Diego County, CA. (World Imagery Basemap source: ESRI, Digital Globe, GeoEye, Earthstar Geographics, CNES/Airbus DS, USDA, USGS, AEX, Getmapping, Aerogrid, IGN, IGP, swisstopo and the GIS User Community).

A member of the family Heteromyidae, the Pacific pocket mouse (PPM, *Perognathus longimembris pacificus*), is one of 16 currently recognized subspecies of the little pocket mouse (*Perognathus longimembris*), and one of the smallest rodents found in North America [16]. Members of the little pocket mouse complex are widely spread throughout arid regions of the United States, extending into northern Baja California peninsula and west-central Sonora, Mexico [16]. Since the 1950’s, PPM have been limited to a handful of distinct populations in southern California (from Los Angeles County to the U.S./Mexico Border), occupying coastal habitat within 4 km of the Pacific Ocean [17]. The PPM was listed as critically endangered in 1994 [17], and extant populations are known in only three locations: Dana Point Headlands (Dana Point, CA), San Onofre, and Santa Margarita (the latter two sites both within the bounds of Marine Corps Base Camp Pendleton [MCBCP], San Diego County, CA, Fig 1). Most of its historic habitat is now destroyed.

Due to its rarity and nocturnal lifestyle, current knowledge of PPM diet is limited. Early dietary studies examined cheek-pouch contents of small numbers of specimens collected near the US-Mexico Border [18] and near Oceanside, California [19]. Later, microscopic fecal analysis of a now extinct PPM population in the San Joaquin Hills, Orange County, CA, identified a seed-dominated diet that is temporally dictated and occasionally includes insects and green vegetation [20]. While that fecal analysis included a larger number of individuals, seeds were rarely identified to genus or species and 73–95% of sample volumes were identified only as grasses or classified as unknown. Given the limitations of these previous observations and studies (e.g., small sample size, poor ability to discriminate plant species) and potential differences in plant communities where PPM are now extant, additional diet analysis is needed to inform conservation management and habitat restoration, especially since much of its remaining habitat has been greatly invaded by non-native annual plants [21] and so may benefit from restoration of native forage.

High-throughput ‘metabarcoding’ techniques have emerged that can classify copies of a taxonomically informative genetic locus persisting in complex mixtures such as feces [14, 15, 22]. These studies amplify representative variants of the barcode locus from the mixture using highly inclusive PCR primers, and then computationally assign those reads to taxa, often by comparing them to a curated database of potential sources [12, 14, 23]. These approaches are attractive because they can work with small amounts of input material, are minimally invasive, have potentially high taxonomic resolution, and recover a high proportion of total dietary components based on rarefaction analysis and comparisons with other observations [24–26]. As PPM fecal pellets are extremely small (~ 2 mm in length and weighing < 1 mg) and contents are difficult to identify by morphological analyses, a metabarcoding approach is appealing.

Here, we describe a metabarcoding analysis of PPM diet using non-overlapping, paired end reads of the internal transcribed spacer (ITS) region of the nuclear ribosomal locus. We assessed potential error and bias in taxonomic assignment with this approach in several ways. First, we evaluated how key bioinformatics choices affected the recovery of “mock” fecal samples of known composition. Second, we used simulations to estimate per-taxon error rates based on an extensive database of the local flora and realistic depictions of sequencing error. Third, we tested our method with captive animals fed a known diet. After performing these evaluations, we examined fecal samples from all three remaining wild populations to determine whether site and seasonal differences in diet composition were detectable. We discuss how this pilot effort has improved our understanding of PPM diet and informs further genetic fecal analysis of PPM and similar herbivores.

## Methods

### Ethics statement

The Pacific pocket mouse is listed as federally endangered by the US Fish and Wildlife Service (USFWS, 2010; 59 FR 5306–5310). The Marine Corps Base Camp Pendleton has committed to the monitoring and management for the endangered PPM as described in the PPM Monitoring Protocol for MCBCP 2011 and the MCBCP Integrated National Resources Management Plan, October 2001. This study was approved by the Western Ecological Research Center Animal Care and Use Committee in association with the University of California, Davis.

### Field collections

PPM fecal pellets were collected directly from captured animals or collected from Sherman live-traps between March and July of 2014 at San Onofre, and two adjacent sites (Oscar One and Edson Training Areas) within the Santa Margarita population. Within the same period, fecal pellets were also collected from track tubes deployed at the same population sites and also at Dana Point Headlands (Fig 1). All Sherman traps and track tubes were baited with sterilized millet seed (*Panicum miliaceum*). Only track tubes containing verified PPM tracks and no other rodent sign were used in this study. Traps were cleaned and track tube cards were replaced between all trap events to ensure there was no cross-contamination of scat from other PPM individuals or other rodent species. All trapping was conducted in accordance with FWS permit TE-045994-14 and California Scientific Collecting permits SCP-838, SCP-4186, SCP-6488 and SCP-7850. Fecal pellets were also obtained from three captive reared PPM individuals fed a controlled diet at the conservation breeding facility at the San Diego Zoo Safari Park. Upon collection, fecal pellets were stored at -20°C until extractions could occur.

In addition to fecal pellets, a total of 26 leaf or tissue samples from plants common within the population sites, as well as those identified in previous PPM diet studies, were collected from habitat within the San Onofre and Santa Margarita populations. This included the millet seed (*P*. *mileaceum*) used to bait the traps and track tubes. Seeds from 13 plant species comprising the seed mix for captive reared animals were also obtained (S1 Table). Captive animals in our study were fed a diet containing a commercial finch seed mixture of oats (*Avena fatua*), flaxseed (*Linum usitatissimum*), canary seed (*Phalaris canariensis*), millet (*Panicum* and *Setaria* spp.) and rapeseed (*Brassica rapa*). The captive diet was supplemented with seeds of native California bunch grass (*Nasella pulchra*), aster (*Corethrogyne filaginifolia*), California sage (*Artemisia californica*), croton (*Croton* sp.) buckwheat (*Eriogonum fasciculatum*), white sage (*Salvia apiana*), and salt grass (*Distichlis spicata*). Captive animals also received romaine lettuce (*Lactuca sativa*) and spinach (*Spinacia oleracea*) leaves as well as mealworms (Coleoptera: Tenebrionidae *Tenebrio molitor*). All seed and plant tissues have been retained at the USGS, Western Ecological Research Center, San Diego Field Station for future analyses.

### DNA extraction and sequencing

#### DNA extraction from fecal samples

Most field samples were extracted as individual pellets. Because fecal pellets are extremely small, we suspected that inter-pellet heterogeneity could be high. Therefore, for a subset of field (N = 3) and captive animals (N = 3) fecal pellets were extracted in sets of 1, 2 or 4 pellets per individual (reflecting the typical range recovered) in order to examine whether pooling pellets at this scale reduces sample heterogeneity.

Pellets were homogenized with a sterile pestle in a 1.5-ml centrifuge tube and then extracted with the Qiagen DNeasy plant kit (Valencia, CA) following manufacturer’s protocols. Random samples of DNA extracts were analyzed on an Agilent 2100 Bioanalyzer using a high-sensitivity assay kit. Fragments in the target amplicon range were usually apparent (albeit not known to be of plant origin), and in the one case where it was not, no qualitative difference in the number or type of species could be discerned for that sample. All samples were stored at -20°C until PCR was performed.

#### Amplification of ITS region

ITS sequencing followed methods described more fully in Cornman et al. [27], in which an approximately 900-bp fragment is subjected to 300-bp paired-end sequencing, recovering non-overlapping fragments of the ITS1 and ITS2 spacer regions. Briefly, amplicons were produced in two steps, first using ‘standard’ primers to generate a high concentration of input template, followed by less efficient ‘fusion’ primers that incorporate exogenous sequencing adapters. The first amplification reaction used primers ITS5a [28] (5’—ACC TTA TCA TTT AGA GGA AGK ARA ART CGT AAC AAG GT—3’) and ITS4 [29] (5’—TTC CTC CGC TTA TTG ATA TGC TTA ARY TCA GC—3’). The thermocycler program consisted of an initial denaturation step of 95°C for 5 min, followed by 40 cycles of 30 s at 95°C, 35 s at 47°C, and 1.5 min at 72°C, and a final extension of 72°C for 10 minutes. An appropriately sized amplification product was confirmed for each reaction by electrophoresis of 5 μL of the reaction product through a 1.5% I.D.NA agarose gel (Cambrex Corporation, East Rutherford, NJ) at 100 V for 45 min. Polymerase chain reaction (PCR) products were cleaned with the Qiagen PCR Purification Kit (Valencia, CA) and quantified using the Qubit dsDNA HS Assay Kit (ThermoFisher Scientific, Grand Island, NY). Samples were diluted in 10 mM Tris buffer (pH 8.5) to a final concentration of 5-ng/μL.

#### Generation of mock fecal samples

To better understand and minimize sources of error or bias in taxonomic assignment, we created two mock fecal extractions by mixing pure sequences from known plant taxa at defined concentrations. For each plant, approximately 25-mg (dry weight) of tissue was ground with a sterile mortar and pestle and the homogenate extracted with the Qiagen DNeasy plant kit (Valencia, CA) following manufacturer’s protocol. ITS sequence was amplified from each species using the same primer-protocol combination described above. A total of 19 PCR products were mixed at equal concentration (mass/volume) to generate mock sample 1 (“Mock 1” hereafter), whereas mock sample 2 (“Mock 2”) consisted of 13 “high” concentration PCR products mixed at 5-ng/μL and 13 “low” concentration PCR products mixed at 1-ng/μL (S1 Table).

To confirm the identity of these inputs, each ITS amplicon was Sanger sequenced from both ends on an ABI3130xl using Big Dye Terminator Cycle Sequencing chemistry (Applied Biosystems, Forster City, CA). Forward and reverse sequences were overlapped and manually edited with Sequencher v5.4 (Gene Codes, Ann Arbor, MI) and deposited in Genbank (Accession numbers KX147515, KX147516, KX147518–147534, KX220084 –KX220090). Some of the inputs were from subspecies that were later determined to be too similar to distinguish by read mapping, and so their expected proportions in the sample were combined (see below).

#### Library prep and quality assessment

Using ITS primers modified with the sequencing adaptors specified in Illumina’s 16S Metagenomic Sequencing Library Preparation (CT #: 15044223 Rev. B), amplicon libraries were prepared following the manufacturer’s protocol. This protocol uses Illumina’s Nextera XT multiplex library indices, which incorporates two distinct 8-bp sequences on each end of the fragment. These indices are read by separate machine cycles (“indexing reads”) that follow the forward 300-bp read and precede the reverse 300-bp read and thus are not part of the analyzed reads themselves. Read pairs are automatically assigned to samples on the basis of these index reads by the MiSeq software. Libraries were diluted 1:10 with molecular grade water and quantified with the Qubit dsDNA HS Assay Kit (ThermoFisher Scientific, Grand Island, NY). DNA size spectra were determined with the Agilent 2100 Bioanalyzer using the Agilent DNA 1000 Kit (Santa Clara, CA). The combined pool of indexed libraries was diluted to 4nM using 10-mM Tris pH 8.5. A final 15-pM preparation was created with a 6.5% PhiX control spike.

#### Read filtering

Machine-processed FASTQ files were imported into CLC Genomics v.7 (Qiagen Bioinformatics Redwood City, CA,) for initial filtering of exogenous sequence adaptors and poor-quality base calls. Adaptors were matched by scanning for regions of similarity to the full-length adaptor reference, using a +2/-3 scoring scheme for a match/mismatch and a minimum score of 10. Degenerate positions in the primer sequences were accommodated by providing multiple explicit variants as search motifs. A maximum error probability of 0.01 was allowed, and a minimum read length of 150 bases was required after all trimming steps. Machine-processed sequencing output has been deposited under BioProject PRJNA339295, SRA accession SRP082705.

### Creation of reference databases

Distinct reference databases were required for analysis of the different sample types. First, the mock fecal samples were compared against the ITS sequences generated with Sanger sequencing from the input plant material. Initial mappings showed significant polymorphism between mapping reads and some of the Sanger-sequenced references, specifically for *Brassica tournefortii*, *Eriogonum fasciculatum* and *Avena* sp., which suggested that our Sanger sequences did not represent the total ITS diversity in the mock samples. Haplotypic variation within the ITS region is not unusual because nuclear ribosomal loci are multi-copy; recent divergence or hybridization in particular can lead to incomplete lineage sorting of ITS copies among related species [30]. To better represent haplotype variants and thus improve the recovery of reads, we added GenBank accessions to supplement the existing references for *Brassica tournefortii* (GQ268069.1) and *Eriogonum fasciculatum* (JQ352513.1). As we continued to see haplotypic variation in reads mapping to *B*. *tournefortii*, we chose three reads as representative of *Brassica*-like variation in the mock sample (all three had strong Blast matches to various Brassicaceae and could not have arisen from other inputs of the mock). To represent variation in reads mapping to *Avena* sp., a consensus sequence of initial mappings was created using samtools [31] and added to the database. The final mock database included 31 sequences representing 21 genera.

The second database represented the diet of the captive population. This database was compiled from ITS sequences isolated from the seed components of the captive diet (mentioned above). The leafy components, spinach (*Spinacia oleracea)* and lettuce (*Lactuca sativa*), were represented by GenBank accessions AB935678.1 and AJ633337.1, respectively.

While the previous databases were developed using the ITS5a + ITS4 primer combination, which produces ITS1 and ITS2 sequence data, for the much larger flora against which field specimens were compared, available ITS2 sequence far exceeded available ITS1 sequence. We therefore limited our analysis of field samples to the ITS2 region only. 1,963 ITS2 Barcode of Life Data Systems (BOLD) accessions (available from www.boldsystems.org; project code SDH), representing approximately 70% of vouchered plant taxa from San Diego County, were downloaded on 24 November 2015. This initial database was then modified by removing accessions designated as hybrids and sequences less than 150-bp in length. Matches to subspecific taxa were summed at the species level. We also added the voucher sequences and two GenBank accessions used in the mock-sample reference database, resulting in 1,966 sequences representing over 1,700 species present in San Diego County. This set included representatives of all 88 plant species previously noted in field studies conducted where PPM occur [32].

Our Sanger-sequenced references varied slightly in completeness due to short reads and quality trimming. This is not unusual when sequencing uncloned PCR product and it was evident in the BOLD ITS2 sequences as well. Small differences in length can introduce a bias in that a higher score may be possible for a read when aligned to the wrong source, simply because that reference overlaps the read to a greater extent than the true, but truncated, reference. We therefore aligned sequences within each of the mock and zoo databases, and trimmed each of these alignments back to a consistent start and stop to help reduce length effects. Alignments were performed with CLUSTALW under default parameters.

### Selection of read-mapping parameters

Bowtie2 [33] was used for all read mapping and samtools [34] for all manipulations of the resulting alignments. Tablet [35] was used to visualize mappings and extract individual reads for exploratory analysis.

In initial alignments of reads to these databases, we observed that mismatches with the ends of the reference sequences, particularly indels in the reads, were not uncommon and some taxa did not map well under end-to-end mode. We therefore chose to use only “local” mapping, which does not require that the full read map but only that a minimum alignment be detected. Local mapping is also more likely to map a chimeric read to a correct parent than end-to-end mapping. Use of a local mapping algorithm potentially traded off specificity for sensitivity, but led to improved recovery of the mock sample proportions (see Results) and its reliability for analyzing the field samples was evaluated by simulation (see below).

For mock and zoo samples, both reads of a pair were informative of mapping biases and rates of chimera formation (the joining of two distinct DNA templates into an artificial molecule during PCR, which can produce erroneous taxonomic assignments), whereas only ITS2 sequence from read2 was informative for taxonomic assignment of field samples. Since a high proportion of chimeras were evident in initial paired-read mappings (see S2 Fig for detailed methods and results), analyses that used both reads treated them as unpaired to reduce the number of reads lost to phylogenetic discordance of the pairs. We also considered the effects of chimera breakpoints occurring within reads in our simulation of taxon-specific error rates, described below, although it seems likely that most chimera breakpoints would lie in the vicinity of the conserved 5.8S portion of the amplicon and thus not be evident in individual reads.

We used the mock samples to guide the choice of scoring threshold used for mapping in Bowtie2. We performed five mappings of both read1 and read2 to the mock reference database, progressively increasing the base component S of the minimum required score to retain a mapping, S + 8.0*ln(L), where ln(L) is the natural logarithm of the read length. The actual score of each read-reference alignment is by default incremented +2 for each match and decremented -5/-3 for opening and extending gaps, respectively, in either reference or read. The default value of S is 20 and, in our estimation, too permissive to use with metabarcoding data, as it would allow a 150-bp read to map successfully under default local parameters if it contains a perfect match of (20+8*ln(150))/2 ≈ 31 bases. We therefore tested higher values in the range of 60–100, in ten point increments. The goodness of fit between observed and expected proportions for each mapping run was assessed as $\;\frac{\left(Obs-Exp\right)2}{Exp}$. Note that while this metric is of the form of the chi-square statistic, the chi-square distribution is based on counts, not proportions, and we are not making any use of the probabilities associated with that distribution. The parameter set resulting in the minimum deviation between observed and expected proportions was then re-evaluated for mappings with read2 only. Based on this evaluation (see Results), a single minimum score threshold was used for all subsequent simulations and analyses.

### Simulation of mapping error

We simulated the rate of erroneous assignments of sequence reads, based on the actual flora of the region and the specific characteristics of our sequencing run. This approach is useful because error in the assignment of reads to the correct source can arise from errors in the sequences themselves [36, 37], the occurrence of chimeric templates during PCR, differing information content of reads of different lengths, varying completeness of reference sequences, and varying distinctiveness of (i.e., genetic distance among) reference sequences. To estimate this error rate per taxon, we simulated reads with realistic levels of error/ambiguity and determined how they would be assigned by the chosen bioinformatics workflow. The simulated database was the same ITS2 database used for field samples with a few minor exceptions, in order to avoid assignment artifacts. First, we removed any provisional or nonstandard taxa lacking a complete Latin binomial name. We also removed any reference sequences that were less than 70% of the length of the longest reference for the corresponding genus, unless no other reference sequence was available. This trimming resulted in 1,899 sequences representing 1,624 species and 667 genera. We used Grinder 0.5.3 [38] to generate realistic sequence reads from this slightly reduced reference database. Reads were simulated with a linearly increasing error rate from 0.1 to 4.0 percent, comparable to the error rate by position estimated by the MiSeq software for the actual sequencing run. The simulated length distribution was taken from the actual filtered reads: a mean ±SD length of 197 ±43 and a minimum length of 150-bp. A range of chimera rates were simulated (1, 10%, 20%, 30%, 40%, 50%) with the minimum kmer requirement disabled.

A likely predictor of assignment error is the presence of heterospecific sequences of high similarity (low genetic divergence). We therefore calculated the smallest genetic distance between any representative sequence of each species in the simulation database to any other heterospecific reference sequence. The pairwise genetic distance matrix was created in Mega6 [39] after first aligning all sequences with Muscle [40] using the default parameters implemented in Mega6 for DNA. The ‘proportional distance’ measure with pairwise deletion of gaps was used, as nucleotide-specific error models were not considered here. The smallest heterospecific genetic distance was identified for each species in the database by looping through the distance matrix with a perl script while referencing a dictionary of conspecific sequences. We then correlated this distance with percent assignment success using Spearman’s rank correlation, implemented with PAST3 [41].

### Taxonomic analysis

Counts of reads assigned to taxa were normalized as counts per million mapped reads. We then excluded from analysis taxa with proportions less than 1% within a sample. We calculated and compared species richness among samples and examined species accumulation curves across all samples for both species and genera to determine whether the sample size adequately captured diet richness. Although imposing a 1% minimum threshold may obscure rare components of the diet composition, and thus underestimate richness, our foremost objective was to describe the more commonly found plant species. Furthermore, there are inherent error rates in DNA amplification, sequencing and read matching which may make it difficult to distinguish low read counts from noise [42, 43]. The Chao2 non-parametric incidence-based richness estimator and 95% confidence intervals [44] were calculated in EstimateS 9.0.1 [45] with 1000 randomizations. Finally, to examine heterogeneity among samples, we compared the Bray-Curtis dissimilarity index among repeated samples from the same individual and among all unique individuals. Zero-adjusted Bray-Curtis dissimilarity based on the standardized frequencies of mapped reads was calculated in Primer 6 [46].

To assess our ability to detect dietary differences among wild populations and between spring and summer seasons, we calculated a zero-adjusted Bray-Curtis dissimilarity index using the genus-level dataset (frequencies >1%) after first removing the genus *Panicum*, which includes the millet seed used as bait (*P*. *milliaceum*). We chose to conduct these analyses with the genus level dataset only, because simulation results suggested greater potential for mapping error among more closely related sequences (see Results below), which are most likely found among conspecifics. Restricting our inferences about site and seasonal differences to the genus-level should constitute a more conservative approach and avoid over-interpretation of what we consider to be a preliminary dataset. Comparisons were made among populations and seasons nested within populations (spring = March-May; summer = June and July) using an ANOSIM (analysis of similarities) with significance assessed with 9,999 permutations of the tests statistic. Dana Point samples were excluded from the nested ANOSIM, as we only obtained samples collected in a single season (spring). We examined which plant species contributed most to differences among populations and seasons using SIMPER (Similarity percentages—species contributions). Analyses were conducted in Primer 6 [46].

### Assessment of unmapped reads

Read2’s from zoo and field samples that were unmapped to their respective libraries were clustered into operational taxonomic units (OTUs) at 95% identity and 80% overlap, using cd-hit-est [47]. Representative reads from each cluster were searched against the nt database (download date 6/15/2016) using BLASTN with the default settings for discontinuous megablast and a minimum bit score of 250 for the highest-scoring pairing (S2 Table).

## Results

### Sequencing output

Total samples consisted of two mock samples, nine fecal samples collected from three captive PPM, and 52 fecal samples collected from wild populations. The total read output was 23.5 million reads in pairs. Initial trimming based on quality, ambiguity, adapter trimming, and a minimum length of 50 resulted in 14.5 million reads. The number of read2’s of 150-bp or greater was 3.49 million for the 52 field samples.

### Simulated mapping error

**Fig 2:**
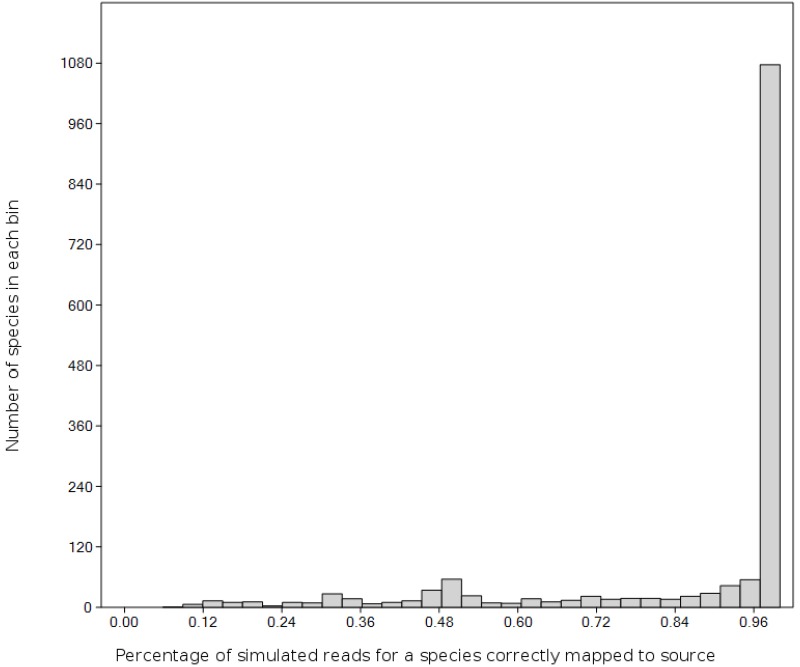
A histogram of simulated matching success. Histogram indicates the proportion of simulated reads mapping correctly to their source for plant species in the simulation reference database and a modeled read-length distribution and error rate. The bins are ranges of percent matching success, chosen using the default method in R. Most species in the simulation database have match-success rates of approximately 97% or greater.

ITS2 generally has among the highest rate of species level assignments when full-length ITS2 regions are available [48, 49]. However, only partial ITS2 sequences were produced by our sequencing strategy and these reads were of variable length (≥150-bp) due to quality trimming. The simulation results showed that most local taxa should nonetheless have high rates of correct assignment (Figs 2 and S2) for a realistic length distribution and error rate. From a total of 1,624 species, simulated reads from 1,254 species were correctly assigned at least 80% of the time, whereas 1,420 species had success rates above 50% (Figs 2 and S3). The minimum genetic distance between a species and its closest relative in the database was strongly correlated with mapping success (Spearman’s r = 0.5835, P = 8.44E-14, N = 1,624), as expected. The mean number of ambiguous reference bases per species also had a weak but still significant relationship (r = -0.0557, P = 0.025, N = 1,624). Changing the rate of chimera formation from zero to 50% had little impact on mapping success rate (S3 Table), showing differentials of a few percentage points.

### Mock fecal sample recovery

**Fig 3:**
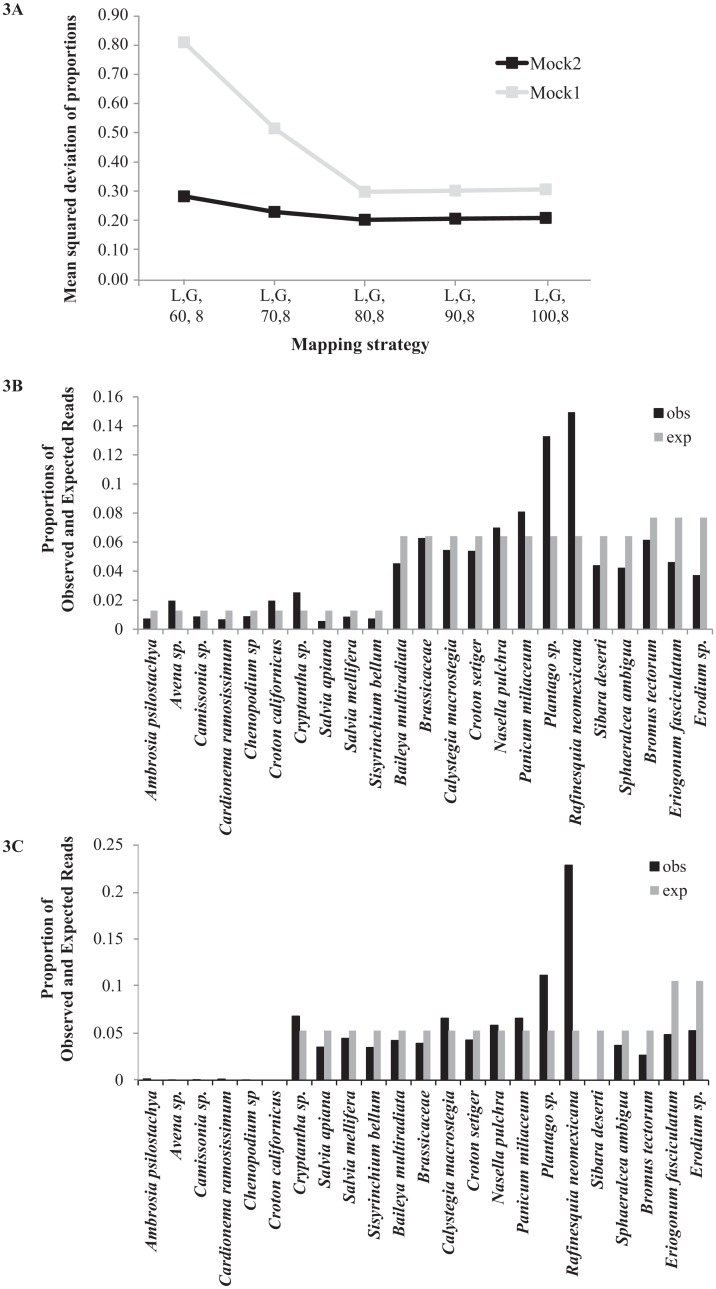
Selection of minimum score parameter for bowtie2 based on known composition of mock communities. (A) Setting the Bowtie2 scoring minimum set to “Local G,80,8”, where 80 is the base score added to the scaling formula, produced mapping proportions with the least deviation from expected $\frac{\left(Obs-Exp\right)2}{Exp}$ for both mock communities, out of the range of values tested. (B) Observed and expected proportions for the Mock1 community. (C) Observed and expected proportions for the Mock2 community.

A threshold mapping score of intermediate stringency most closely recovered the expected proportions for each mock fecal sample. The summed deviations from expected proportions ranged from 0.2 to 0.8 when the base score of the scoring algorithm ranged from 60–100 (Fig 3A). The minimum deviation from expected for both mock samples was for a base score of 80 when mapping both read1 and read2 singly. Mapping only with read2, deviations between expected and recovered proportions were very similar for parameter values in the range of 70 to 90 (data not shown) and lower than other tested parameter values. We therefore chose a base threshold score of 80, which recovered most species in proportions similar to that expected in the mock samples (Fig 3B and 3C). *Plantago* and *Rafinesquia* were most consistently over-represented whereas *Salvia* species (*S*. *apiana* and *S*. *mellifera*) were consistently under-represented, and these biases were persistent across the range of threshold scores investigated. The over-representation of *Plantago* may have been related to its frequent participation in chimeras, as measured by read-pair discordance (S1 Fig).

### Captive Sample Assignments

**Fig 4:**
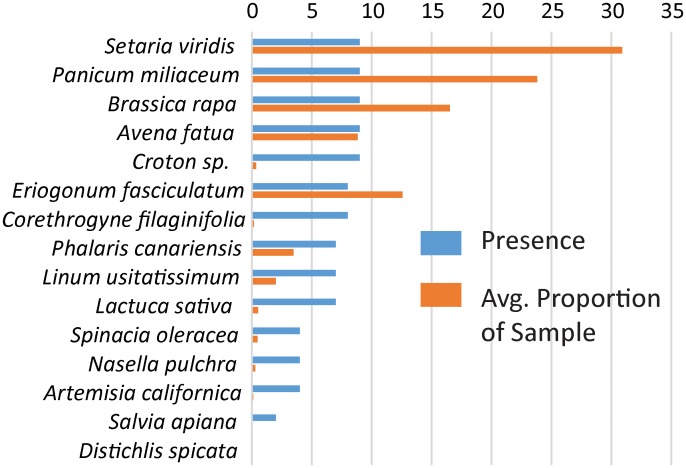
Average proportions of mapped reads (orange bars) and total number of fecal samples (blue bars) in which plant species were detected in captive reared PPM fecal pellets.

We recovered all plant species in the diet of captive animals except salt grass (*D*. *spicata*; Fig 4 and S4 Table). White sage (*Salvia apiana*) was recovered in extremely low proportions (0.001% overall), however. The proportions of reads mapping to taxa varied among sample pools of one, two, or four pellets taken from the same individual, often dramatically so. While pooling pellets did not appear to increase the overall number of taxa recovered (S4 Table), any quantitative model of diet based on count data will need to consider inter-pellet heterogeneity.

### Field Sample Assignments to SD ITS2 Database

**Fig 5:**
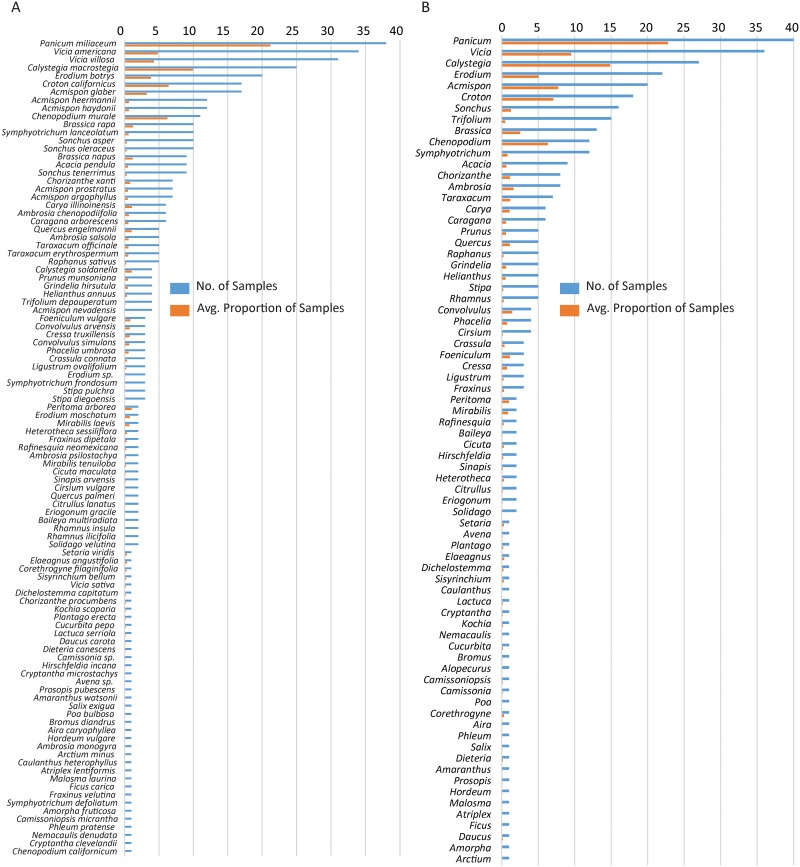
Average proportion of mapped reads (orange bars) and total number of fecal samples (blue bars) in field collected PPM fecal pellets. This value is given for (A) plant species, and (B) plant genera recovered.

A total of 839,443 reads from field samples mapped to the reference ITS2 Database (mean 16,454 ± 9,356 SD per sample). In total, 412 plant species were assigned reads, 111 of which met a 1% minimum detection threshold in at least one sample (Fig 5A). When aggregated at the genus level, 74 plant genera were recovered at proportions of >1% in the field collected fecal samples (Fig 5B; S5 Table). The majority of these were forbs (66%), 10 were grasses (14%), and the remaining genera were trees and shrubs (20%). Most recovered genera (94%) had estimated error rates by simulation of <10% (S5 Table). The genus *Panicum* was recovered in the highest number of fecal samples (39 of 52), followed by *Vicia*, *Calystegia*, *Erodium*, *Acmispon*, *Croton*, *Sonchus*, *Trifolium*, *Brassica*, and *Chenopodium* (Fig 5B; S5 Table).

**Fig 6:**
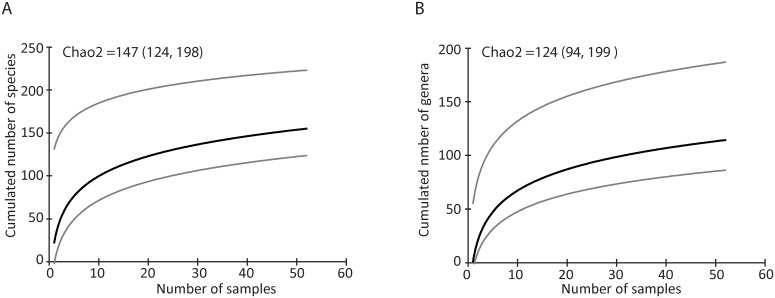
Species richness accumulation curves. Showing (A) the estimated number of plant species and (B) the estimated number of plant genera in increasingly pooled samples of field collected fecal pellets. Mean values and 95% confidence intervals are shown for 100 replicates.

Taxon accumulation curves did not reach a clear asymptote at either species or genus rank (Fig 6A and 6B). Chao2 estimators were greater than observed (species Chao2 mean = 147, 95% CIs: 124,198; Genera Chao2 mean = 124, 95% CIs: 94,199), suggesting that overall diet richness is underestimated with 52 samples. This likely reflects low per-sample richness coupled with high variability among samples. Plant genera richness ranged from four to 14 per sample. In addition, there was substantial heterogeneity among fecal pellets collected at the same time from the same individual in both captive and field samples (average Bray-Curtis Index = 0.3915, SD = 0.2884, range 0.0206–0.8257, 24 comparisons; S4 and S5 Tables). This distribution overlapped with those recovered across unrelated samples (average Bray-Curtis Index = 0.2137, SD = 0.1876, range 0.01–0.8829, 1806 comparisons).

In the one-way analysis of similarity among all three populations, the global test showed no differences among all three sites (R = 0.102, p = 0.071). However, in pairwise comparisons, Santa Margarita and Dana Point differed significantly in composition (R = 0.204, p = 0.041). The pairwise SIMPER showed that Santa Margarita and Dana Point had an average dissimilarity of 88, with over 50% of this driven by higher average relative abundance of *Calystegia* and *Vicia* in Santa Margarita and higher average relative abundance of *Acmispon* and *Croton* in Dana Point (Table 1). In the analysis of similarity with season nested within population (excluding Dana Point where only spring samples were collected), we detected a significant difference in composition between seasons (R = 0.136, p = 0.008), but not among populations. Spring and summer had an average dissimilarity of 82, which reflected higher spring average relative abundances of *Chenopodium* and *Erodium*, and higher summer average relative abundances of *Calystegia*, *Vicia* and *Croton* (Table 2).

**Table 1:** Similarity analysis (SIMPER) among populations, showing differences in genera abundance between Santa Margarita (N = 37) and Dana Point Headlands (N = 5) with the average relative abundance (Avg abundance), average dissimilarity along with standard deviation (Avg Dissimilarity ± SD), percent (%) contribution, and percent cumulative.

Genus	Santa Margarita Avg Abundance	Dana Pt Headlands Avg Abundance	Avg Dissimilarity ± SD	% Contribution	% Cumulative
*Acmispon*	2.82	40.39	19.63 ± 1.15	22.28	22.28
*Calystegia*	25.84	1.63	12.80 ± 0.86	14.52	36.8
*Vicia*	12.19	11.44	7.56 ± 1.05	8.58	45.38
*Croton*	5.84	12.07	7.39 ± 0.85	8.38	53.76
*Chenopodium*	9.56	7.05	6.99 ± 0.74	7.93	61.69
*Mirabilis*	0	9.18	4.59 ± 0.71	5.21	66.91
*Erodium*	9.01	0.36	4.50 ± 0.57	5.1	72.01
*Brassica*	4.09	0.91	2.29 ±0.52	2.6	74.61
*Acacia*	0.21	3.85	1.91 ± 1.05	2.17	76.78
*Chorizanthe*	2.36	1.52	1.75 ± 0.45	1.99	78.76
*Ambrosia*	2.32	1.3	1.64 ± 0.50	1.87	80.63
*Heterotheca*	0	2.73	1.36 ± 0.50	1.55	82.18
*Carya*	2.46	0	1.23 ± 0.29	1.4	83.57
*Symphyotrichum*	1.11	1.9	1.14 ± 0.94	1.29	84.87
*Convolvulus*	2.21	0	1.10 ± 0.25	1.25	86.12
*Sonchus*	1.31	1.62	1.08 ± 1.23	1.22	87.34
*Quercus*	2.01	0	1.01 ± 0.27	1.14	88.48
*Rafinesquia*	0.16	1.68	0.89 ± 0.53	1.01	89.48
*Grindelia*	0.31	1.12	0.66 ± 0.55	0.75	90.23

Average Dissimilarity = 88.1.

**Table 2:** Similarity analysis (SIMPER) between seasons. Showing differences in genera abundance between springs (N = 27) and summer (N = 21) sampling periods nested within site and with the average relative abundance (Avg Abundance), average dissimilarity along with standard deviation (Avg Dissimilarity ± SD), percent (%) contribution, and percent cumulative.

Genus	Spring Avg Abundance	Summer Avg Abundance	Avg Dissimilarity ± SD	% Contribution	% Cumulative
*Calystegia*	18.93	26.55	15.18 ± 1.10	18.48	18.48
*Vicia*	12.9	16.19	9.05 ± 1.09	11.01	29.49
*Chenopodium*	13.02	1.21	6.82 ± 0.56	8.3	37.79
*Erodium*	13.35	1.85	6.69 ± 0.70	8.14	45.93
*Croton*	4.43	10.86	6.58 ± 0.67	8.01	53.94
*Acmispon*	5.37	4.05	3.91 ± 0.69	4.76	58.7
*Brassica*	4.63	2.96	3.30 ± 0.61	4.02	62.72
*Carya*	2.46	1.93	2.04 ± 0.42	2.48	65.2
*Convolvulus*	0.04	3.89	1.96 ± 0.35	2.38	67.58
*Chorizanthe*	0.87	3.08	1.89 ± 0.36	2.3	69.88
*Ambrosia*	2.92	0.71	1.72 ± 0.44	2.1	71.97
*Taraxacum*	0.1	3.24	1.64 ± 0.36	2	73.97
*Phacelia*	2.27	0.67	1.43 ± 0.28	1.75	75.72
*Quercus*	2.8	0.07	1.43 ± 0.33	1.74	77.45
*Caragana*	0	2.58	1.29 ± 0.43	1.57	79.02
*Peritoma*	0.22	2.25	1.22 ± 0.24	1.49	80.51
*Foeniculum*	2.1	0.24	1.15 ± 0.24	1.4	81.91
*Sonchus*	1.65	1.14	1.13 ± 0.74	1.38	83.29
*Cressa*	0	1.95	0.98 ± 0.35	1.19	84.48
*Symphyotrichum*	0.62	1.5	0.92 ± 0.70	1.12	85.61
*Helianthus*	0.06	1.77	0.90 ± 0.30	1.1	86.71
*Prunus*	0.84	0.49	0.63 ± 0.39	0.77	87.47
*Crassula*	1.2	0	0.60 ± 0.28	0.73	88.2
*Trifolium*	0.51	0.91	0.56 ± 0.86	0.68	88.88
*Elaeagnus*	0	1.07	0.54 ± 0.22	0.65	89.54
*Raphanus*	0.27	0.68	0.45 ± 0.44	0.55	90.09

Spring and Summer Average dissimilarity = 82.16.

## Discussion

This study investigated the use of a genetic barcode to identify plant species consumed by PPM. In the only previous quantitative study of PPM diet, from a now extinct population in Orange County, Meserve [20] identified six plants to the rank of genus or species in the diet of PPM using microscopy: California buckwheat (*Eriogonum fasciculatum*), deerweed (*Ascmispon glaber*), lemonadeberry (*Rhus integrifolia*), sage (*Salvia sp*.), storksbill (*Erodium sp*.), and Cleveland’s cryptantha (*Cryptantha clevelandii*). We recovered all of these genera in our study, although some (particularly *Salvia* and *Rhus*) were recovered in low proportions (<1%). The most prevalent plant species identified in PPM diets in our study included morning glory (*Calystegia macrostegia*), vetch (*Vicia* spp.), storksbill filaree, croton (*C*. *californicus*), deerweed, mustard (*Brassica* spp.), goosefoot (*Chenopodium murale*), sow thistle (*Sonchus* spp.), clover (*Trifolium* spp.), and aster (*Symphotrichum* spp.). We cannot make direct comparisons between our study and Meserve (20), as differences in prevalence may be attributed to varying degrees of bias and resolution in the two methods as well as differences in plant communities among sites. Generally, however, the higher level of richness observed in our study is concordant with other work that found much higher diet richness and greater resolution with next generation sequencing techniques when compared to histological analyses of the same samples [50–52]. Even so, our results suggest that we have underestimated diet richness based on species accumulation curves. Perhaps not surprisingly, we detected the bait seed most frequently in terms of number of fecal samples and the proportion of sequence reads. Although high abundance could also be due to amplification or copy-number bias, *P*. *miliaceum* is available to PPM as bait throughout their active seasons due to continuous population monitoring efforts with track tubes and intermittent live-trapping [32, 53]. Although *P*. *miliaceum* and other native *Panicum* species do occur in San Diego County, these are not prevalent at any of the study sites. Therefore, we assume that the *Panicum* we recovered is from bait and does not demonstrate a strong ecological interaction between these two species.

We detected some site and temporal differences in diet composition, driven by differences in the more frequently detected plants rather than in the rarer diet components. Food selection in generalist herbivores may be driven by a combination of resource availability, nutritional value, foraging energetics costs, and risk aversion [54–58]. Other heteromyid rodents have been shown to select and ingest seeds that are the richest in energy of those available in the environment, as well as seeds with high nutrient and water content [59, 60]. Our results provide a valuable baseline of plant utilization that is consistent with phenology and suggests a broad spectrum of potential food sources, including both native and non-native species. The seasonal differences we detected could reflect different seed and plant availability rather than differences in preference, as the plant genera that most contribute to seasonal variation exhibit different phenologies. For example, the two most commonly detected species of *Erodium* (*E*. *botrys* and *E*. *moschatum*) are annual herbs that emerge and bloom in early spring, while *Calystegia macrostegia*, *Vicia* spp. and *Croton californicus* typically bloom from late spring through June or July [61]. Site differences may reflect differences in plant presence or abundance at Dana Point and Santa Margarita as well. Dana Point is dominated by shrubs, which may explain why deerweed is a larger component of the PPM diet at this site. Although forb cover is low, croton is one of the most abundant forbs at this site and was also more prevalent in the PPM diet. In contrast, Santa Margarita is dominated by forbs and grasses (native and non-native) with lower shrub cover and a more limited distribution of *Croton californicus*. Over all sites, forbs dominated the diet analysis results. Historical descriptions of the flora indicate there were mostly wildflower fields (forb lands) in these regions in the past with dry grasses present in the summer, even prior to the introduction of European invasive species [21].

We found high variability in plant taxa among fecal pellets collected from the same individual at the same time. This heterogeneity may reflect patterns of consumption, differential digestion/degradation, and/or a lack of homogenization during passage. Digestion pass-through rates, for example, can be quite rapid in other rodent species and experimental studies have reported rates ranging from 0.5–13 hours [51, 52]. Soininen et al. [50] reported high variability in recovered diet components among individual fecal samples from two small voles (*Microtus oeconomus* and *Myodes rufocanus*), and high fecal-sample variability has been reported elsewhere [62, 63]. Nonetheless, we expect population-level comparisons based on large samples to be robust to intra-individual noise. For most comparisons, aggregate samples were relatively large given the pilot nature of the study, yet our representation of the Dana Point Headlands population must certainly be considered preliminary. Additionally, we did not attempt to partition the technical from biological components of heterogeneity, such that the importance of random PCR artifacts versus systematic effects (e.g., taxon-specific dropout) remains to be determined. Pooling additional technical replicates prior to sequencing should help minimize random PCR effects, whereas pooling multiple fecal pellets when available will help minimize biological heterogeneity [64].

While useful, fecal metabarcoding is not without complications. General issues include the possibility of PCR inhibition, DNA degradation, and taxonomic biases in effective copy number. Barcode choice is a balance of considerations including fragments sizes recovered, desired taxonomic resolution and specificity, and the availability of suitable reference databases. The ITS primers that we used have pros and cons in these regards. The amplicons are long and thus may amplify poorly on degraded DNA, and will have increased susceptibility to dropout bias (differential decay of DNA from different taxa). Although fragment-size analysis of DNA extracts showed good representation of high-molecular weight DNA, we cannot demonstrate that plant taxa were equally represented in that fraction. Moreover, the primers amplify other taxa in addition to plants, particularly fungi, and thus have lower yield per unit of sequencing effort. Nonetheless, the ITS gene region has been shown to perform well in discriminating plant species relative to other loci [49]. As our primary interest was taxonomic resolution, there was an apparent need for both ITS1 and ITS2 sequence to achieve this resolution at the time this study was initiated. Since then, efforts to voucher sequence the San Diego Natural History Museum’s synoptic collection has provided a rich ITS2 resource but without matched ITS1 data. Thus, for this region and study organism, ITS2 alone is likely a more efficient barcode going forward.

We assessed the reliability and specificity of our metabarcode approach in several ways. First, simulations of matching success using incomplete, variable-length, and possibly chimeric fragments of the ITS2 region indicated high specificity for most species in the database. Second, mock communities were generally recovered in similar proportions to their starting concentrations when using a mapping approach tolerant of mismatches at the ends of reads and of chimeras. Finally, almost all known plant species fed to captive PPM were recovered from their fecal pellets. The biases we did observe may have been due to differences in primer specificity or taxon-specific patterns of chimera formation. For example, observed proportions of *Salvia*, *Eriogonum* and *Erodium* were approximately half that expected in the mock communities. *S*. *apiana* was also poorly recovered in captive PPM fecal pellets. Therefore, we may expect *Salvia* species to be under-represented in field samples. *Plantago* was over-represented in the mock-communities and was highly represented among evident chimeras as well (S1 Fig). None of these biases could have been predicted *a priori*, and we have no direct recourse for detecting biases in taxa present only in field samples. Our approach to minimizing these biases has been to assess multiple realistic communities of known character and then make bioinformatics choices that are most appropriate in that light. Additional mocks and other control samples can be analyzed as knowledge of diet components evolves, to further identify and correct for such biases.

Another complication of barcode-based methodologies is the selection of a suitable reference database. Regional databases might be difficult to construct from available resources, and often need to be supplemented for particular objectives, as was done here. For example, over 1,700 species were represented in our ITS2, but even so approximately 30% of known species in this floristically rich region were unavailable. On the other hand, including species present in a broader region without regard to habitat can increase assignment error, if closely related but ecologically implausible congeners are included. This effect was evident in the strong positive correlation we found between assignment error rates and genetic similarity to other taxa in the simulation results. While we chose to conservatively include all plant taxa presently available from BOLD for San Diego County, rather than limiting our ITS2 database to the much smaller (but possibly incomplete) list of plants documented at the study sites, future work may include further refinements of the database.

The majority of plant species identified in the diet of wild-caught PPM have been identified to at least the genus level within PPM population boundaries during habitat surveys associated with annual monitoring (Brehme et al. 2010). One notable exception is vetch (*Vicia* spp.) which was the second most prevalent genus identified in the PPM scat but has never been recorded during annual habitat surveys for thousands of subplots. However, only the top 3 to 4 dominant forb species per subplot are recorded during these surveys and both vetch species have been independently documented to occur on Marine Base Camp Pendleton (SDNHM Database of San Diego County Collected Plant Specimens, accessed 29 February 2016). Therefore, vetch may well be present but not a dominant member of the forb community, available in the seed bank, a contaminant in the bait seed, or possibly be misclassified but a closely related species in the Fabaceae family. More detailed plant surveys and diet experiments will help to determine the presence of such plant species within the study area, as well as inform the detection threshold and reference database as needed for future resource selection studies.

In conclusion, ITS metabarcoding effectively identified food sources of an endangered, elusive animal, using minute inputs gathered with minimal harm. Metabarcoding had higher resolution than microscopy and potentially lower observer bias than microscopy, based on comparisons to early work that sacrificed animals to directly examine pouch or gut contents [19, 20]. The data can inform further conservation and restoration of PPM through its application in a wide array of resource-selection studies and can help to better characterize the relationship between habitat/resource availability and PPM demography. From these preliminary data, several plants appear prevalent in the current diet whereas a much larger diversity was detected at lower levels. PPM likely use both native and non-native plant food sources and adjust their diet according to local and seasonal differences in plant species availability. In addition to any future refinements in the methodology discussed, a larger sample size that better represents the spatial variability in resources, as well as temporal variability of seeds within and among years will be needed to identify plant species most preferred by PPM, and better understand how these preferences may shift with fluctuations in resource availability. Results from a more comprehensive diet and resource selection analyses could then be combined with information such as habitat occupancy, localized colonization and extinction dynamics, and reproduction and recruitment dynamics. The last would be used to help determine if specific food plants are associated with PPM habitat suitability and fitness. Identification of these important food plants will help us to determine the most beneficial plant community for use in managing and restoring habitats for this critically endangered species. An ancillary benefit of genetic fecal analysis that we have not yet explored is the possibility of amplifying markers informative for PPM population genetics, such as microsatellites or SNPs, from the DNA extracts.

## Supporting Information

S1 Fig Methods and results for chimera detection in read pairs. (DOCX)

S2 Fig A phylogram produced with MEGAN [1] representing the relative read-matching success for San Diego-area plant species based on simulated data (see text). Taxa are color-coded based on bins of percent correct mappings: green = 95–100%, blue = 90–94.9%, yellow = 80–89.9%, orange = 50–79.9%, and red = <50%. Variation in the size of labelled nodes is not meaningful in this context and is used only to further discriminate different values in the figure. A few taxa are not represented in the MEGAN taxonomic scheme at the species level and are therefore shown at the generic level instead. The plant genera *Abronia* and *Polypogon* were removed from the phylogram because these taxonomic names are not unique in the taxonomic scheme and are placed within Bilateria by MEGAN. The following species are not recognized by the MEGAN taxonomic scheme: *Apiastrum angustifolium*, *Cardaria chalepensis*, *Dicoria canescens*, *Hutchinsia procumbens*, *Kopsiopsis strobilacea*, *Malperia tenuis*, *Meconella californica*, *Meconella denticulata*, *Poteridium annuum*, *Poterium sanguisorba*, *Bromidium tandilense*, *Cardaria draba*, *Ancistrocarphus filagineus*.
(PNG)

S3 Fig Simulation match success rates to the correct species and genus for 1,624 plant species present in San Diego County. (DOCX)

S1 Table 26 field collected plant species and 15 plant species fed in the diet of captive reared animals for which sequences were generated or obtained. All samples were keyed to genus or species based on morphological characteristics. Two mock samples were created with different proportions of PCR product from field collected plant species. Mock sample 1 (Mock 1) was created with a total of 19 PCR products mixed at equal concentrations (mass/volume). Mock sample 2 (Mock 2) consisted of 13 “high” concentration PCR products mixed at 5ng/ μL and 13 “low” concentration PCR products mixed at 1ng/μL.
(XLSX)

S2 Table Expected level of taxonomic coverage for ITS2 primer that failed to map to reference databases. Only nt accession with bit scores of 250 or more are shown.
(XLSX)

S3 Table Minimal impact of chimera rate on simulated matching success for the San Diego ITS2 simulation database. The difference between the lowest matching-success rate under any of several simulated chimera rates (10, 20, 30, 40, or 50%) and when chimera rates were set to zero is plotted on the horizontal axis versus on the vertical axis the simulated matching-success rate itself when chimera rates were set to zero.
(ODS)

S4 Table Table depicting the mapped read counts from three samples from each of three captive reared zoo animals compared to a sequence database of presented food items. Samples were comprised of 1, 2 or 4 fecal pellets. Plant species are arranged in order from most to least prevalent overall.
(DOCX)

S5 Table Table including the plant genera present at greater than 1% proportion in samples across and within each field site. Plants are listed from most to least prevalent across all samples. The average proportion across samples and the simulation match percentages for each genus are also presented.
(XLSX)
