# Supplementary material for: Metabarcoding of Fecal Samples to Determine Herbivore Diets: A Case Study of the Endangered Pacific Pocket Mouse
Source: PLoS One. 2016 Nov 16;11(11):e0165366. doi: 10.1371/journal.pone.0165366 (PMC5112926; doi:10.1371/journal.pone.0165366)
Supplement: S1 Fig — (DOCX) [file pone.0165366.s001.docx]

**S1 Fig Paired-read mapping to mock communities indicate high but taxonomically biased rates of chimera formation**

Chimera rates were investigated by comparing read1 and read2 assignments using an adjacency matrix approach, as implemented in the R package sna (1). The adjacency matrix was based on the observed frequencies of pairs of taxa that read1 and read2 map to, assuming a non-chimeric read has the same taxon mapping for both reads and a chimeric one does not.

The apparent rate of chimera formation was high in the mock samples. The average rate in which the best-matched taxon of each read in a pair differed, i.e. was chimeric, was 49.7% after lumping related references as noted above. High observed chimera rates may reflect the fact that all amplicons share the conserved 5.8S region, even though that part of the amplicon is not recovered in the reads.

There were evident biases in terms of the pairing of taxa in chimeras (figure), with *Plantago* sp. being the most frequent participant. These results support the use of single rather than paired mapping to reduce the occurrence of phylogenetic discordance. It remains possible that chimera breakpoints will occur within single reads, with the following outcomes: the read will be assigned to one of the parent templates, the read will be assigned to an incorrect source, or the read will be unmapped. The simulation of ITS2 mapping error to the local flora included chimeras occurring within read2’s at rates up to 50% and indicated that chimera breakpoints within read2 had minimal effect on assignment accuracy under local scoring.


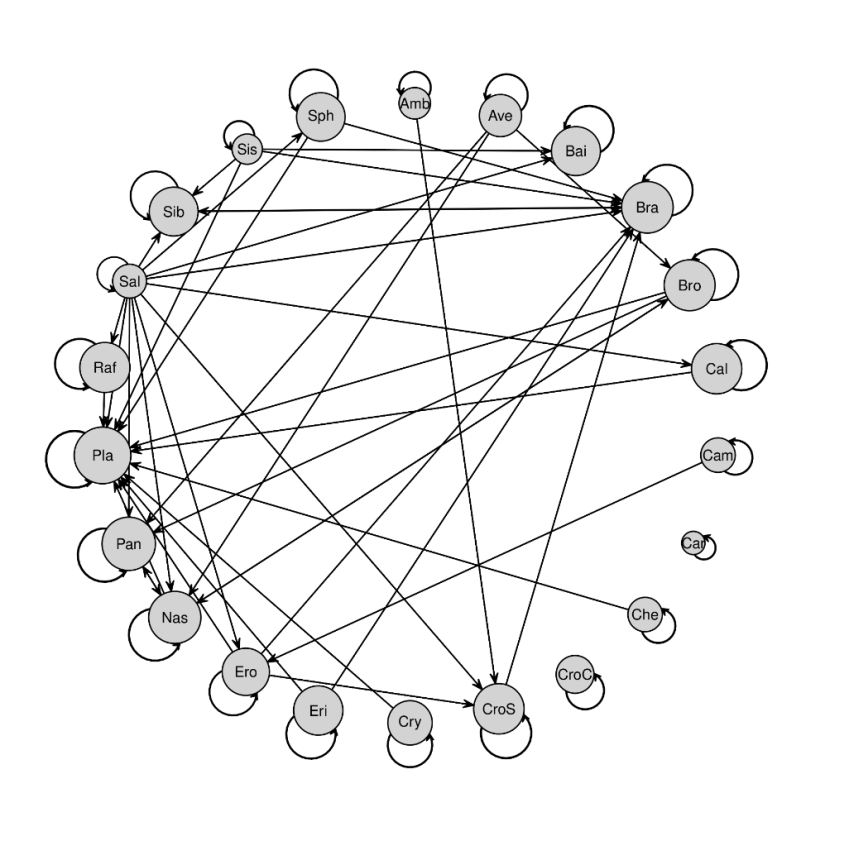


**SF 2 Fig 1.** Adjacency matrix for mock2 showing the relative rate that read1 and read2 of the same pair mapped to the same taxon versus different taxa. Directed arrows pointing between taxa show the proportion of read1s mapped to the source node that were paired with a read2 mapped to another taxon. Arrows that point back to the same node indicate concordant mappings of both reads in a pair. The area of each node is proportional to the total number of reads mapped for mock2 (read1 + read2). Figure created with the R package *sna*. Species codes are translated as follows: Amb (*Ambrosia psilostachya*), Ave (*Avena* spp.), Bai (*Baileya* sp.), Bra (*Brassica* spp.), Bro (*Bromus madritensis*), Cal (*Calystegia* sp.), Cam (*Camissonia* sp.), Car (*Cardionema* sp.), Che (*Chenopodium* sp.), CroC (*Croton californicus*), CroS (*Croton setiger*), Cry (*Cryptantha sp*.), Eri (*Erigonum fasciculatum*), Ero (*Erodium* spp.), Nas (*Nasella cernua*), Pan (*Panicum* sp.), Pla (*Plantago* sp.), Raf (*Rafinesquia* sp.), Sal (*Salvia* sp.), Sib (*Sibara* sp.), Sis (*Sisyrinchium* sp.), Sph (*Sphaeralcea* sp.).

**Literature Cited**

1. Butts CT. sna: Tools for social network analysis R Package Version 2010.
