# Supplementary material for: Metabarcoding of Fecal Samples to Determine Herbivore Diets: A Case Study of the Endangered Pacific Pocket Mouse
Source: PLoS One. 2016 Nov 16;11(11):e0165366. doi: 10.1371/journal.pone.0165366 (PMC5112926; doi:10.1371/journal.pone.0165366)

**S3 Fig** Simulation match success rates to the correct species and genus for 1624 plant species present in San Diego County.


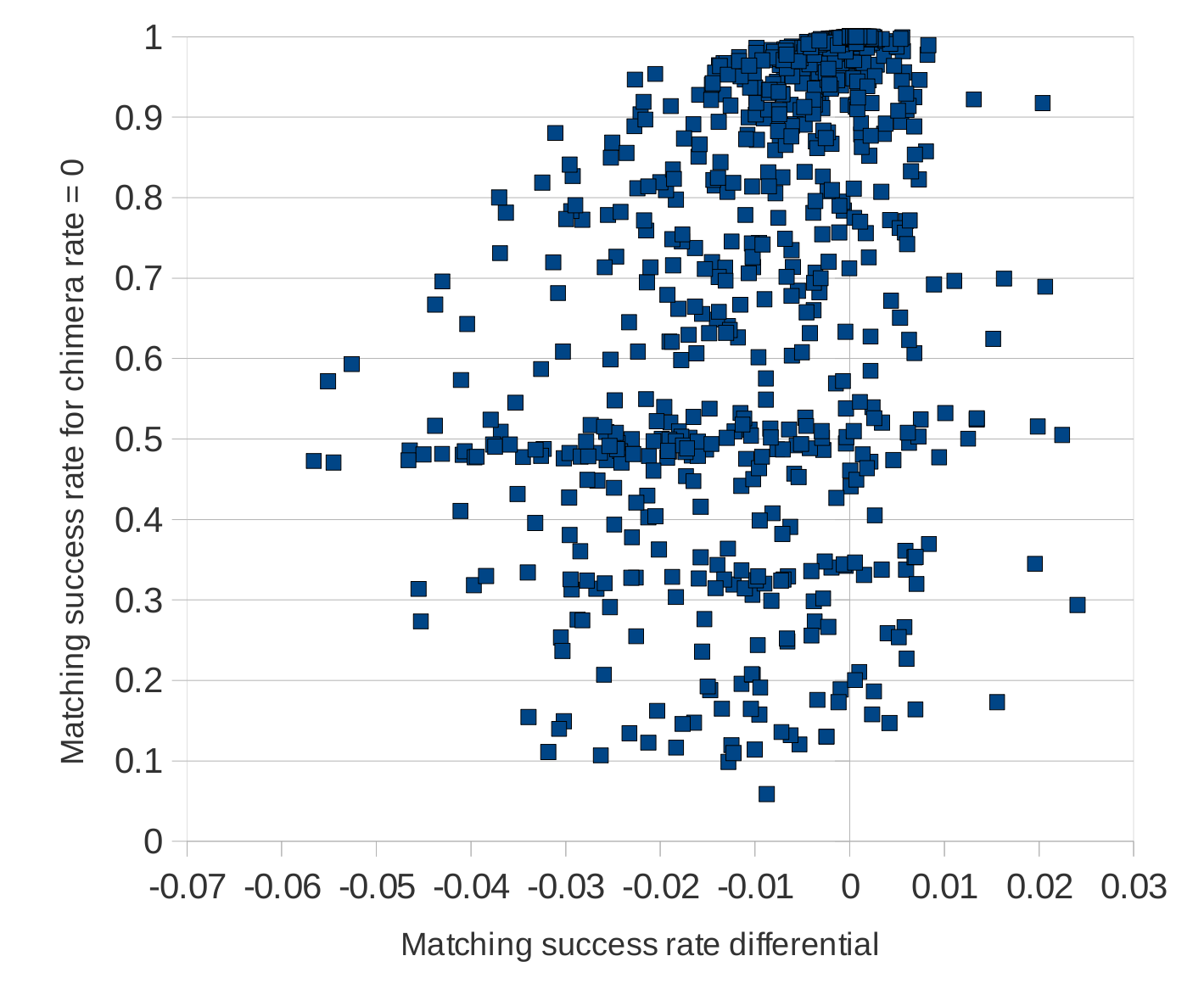

Supplement: S3 Fig — (DOCX) [file pone.0165366.s003.docx]
