# Supplementary material for: Metabarcoding of Fecal Samples to Determine Herbivore Diets: A Case Study of the Endangered Pacific Pocket Mouse
Source: PLoS One. 2016 Nov 16;11(11):e0165366. doi: 10.1371/journal.pone.0165366 (PMC5112926; doi:10.1371/journal.pone.0165366)
Supplement: S4 Table — Samples were comprised of 1, 2 or 4 fecal pellets. Plant species are arranged in order from most to least prevalent overall. (DOCX) [file pone.0165366.s007.docx]

| **S4 Table** Relative proportions of mapped read counts from three samples from each of three captive reared zoo animals compared to a sequence | | | | | | | | | |
| --- | --- | --- | --- | --- | --- | --- | --- | --- | --- |
| database of presented food items. Samples were comprised of 1, 2 or 4 fecal pellets. Plant species are arranged in order from most to least | | | | | | | | | |
| prevalent overall. | | | | |  |  |  |  |  |
| **Reference** | **Zoo1-1** | **Zoo1-2** | **Zoo1-4** | **Zoo25-1** | **Zoo25-2** | **Zoo25-4** | **Zoo30-1** | **Zoo30-2** | **Zoo30-4** |
| *Setaria viridis* | 0.10 | 94.40 | 78.60 | 14.40 | 22.80 | 10.10 | 17.90 | 19.50 | 20.30 |
| *Panicum miliaceum* | 69.10 | 2.00 | 2.00 | 81.80 | 2.60 | 3.70 | 47.40 | 5.50 | 0.10 |
| *Brassica rapa* | 0.10 | 0.00 | 11.40 | 1.10 | 5.20 | 84.90 | 10.50 | 31.00 | 4.30 |
| *Eriogonum fasciculatum* | 0.00 | 0.00 | 3.20 | 1.80 | 51.20 | 0.00 | 16.40 | 26.40 | 14.20 |
| *Avena fatua* | 28.30 | 0.00 | 0.50 | 0.50 | 0.10 | 0.00 | 0.10 | 0.20 | 49.80 |
| *Phalaris canariensis* | 0.00 | 0.00 | 0.00 | 0.10 | 0.10 | 0.10 | 7.40 | 13.70 | 9.90 |
| *Linum usitatissimum* | 0.00 | 0.00 | 0.00 | 0.00 | 17.80 | 0.10 | 0.00 | 0.00 | 0.00 |
| *Lactuca sativa* | 0.00 | 0.00 | 0.00 | 0.00 | 0.00 | 0.00 | 0.00 | 3.40 | 1.30 |
| *Spinacia oleracea* | 0.00 | 0.00 | 4.30 | 0.00 | 0.00 | 0.00 | 0.00 | 0.00 | 0.00 |
| *Croton sp.* | 0.00 | 2.80 | 0.10 | 0.10 | 0.10 | 0.00 | 0.00 | 0.10 | 0.00 |
| *Nasella pulchra* | 2.40 | 0.00 | 0.00 | 0.00 | 0.00 | 0.00 | 0.00 | 0.00 | 0.00 |
| *Corethrogyne filaginifolia* | 0.00 | 0.70 | 0.00 | 0.00 | 0.10 | 0.10 | 0.20 | 0.30 | 0.00 |
| *Artemisia californica* | 0.00 | 0.00 | 0.00 | 0.00 | 0.10 | 0.90 | 0.00 | 0.00 | 0.00 |
| *Salvia apiana* | 0.00 | 0.00 | 0.00 | 0.00 | 0.00 | 0.00 | 0.00 | 0.00 | 0.00 |
| *Distichlis spicata* | 0.00 | 0.00 | 0.00 | 0.00 | 0.00 | 0.00 | 0.00 | 0.00 | 0.00 |
